# Digital Gaming for Improving the Functioning of People With Traumatic Brain Injury: Randomized Clinical Feasibility Study

**DOI:** 10.2196/jmir.7618

**Published:** 2018-03-19

**Authors:** Maritta Välimäki, Kaisa Mishina, Johanna K Kaakinen, Suvi K Holm, Jukka Vahlo, Markus Kirjonen, Virve Pekurinen, Olli Tenovuo, Jyrki Korkeila, Heikki Hämäläinen, Jaana Sarajuuri, Pekka Rantanen, Tage Orenius, Aki Koponen

**Affiliations:** ^1^ Department of Nursing Science Faculty of Medicine University of Turku Turku Finland; ^2^ Turku University Hospital Turku Finland; ^3^ School of Nursing Hong Kong Polytechnic University Hong Kong China (Hong Kong); ^4^ Department of Psychology Faculty of Social Sciences University of Turku Turku Finland; ^5^ Turku School of Economics Centre for Collaborative Research University of Turku Turku Finland; ^6^ Division of Clinical Neurosciences Turku University Hospital Turku Finland; ^7^ Faculty of Medicine University of Turku Turku Finland; ^8^ Psychiatric Care Division Satakunta Hospital District Harjavalta Finland; ^9^ Validia Rehabilitation Helsinki Helsinki Finland; ^10^ Orton Orthopaedic Hospital Orton Helsinki Finland

**Keywords:** rehabilitation, traumatic brain injury, video games

## Abstract

**Background:**

Traumatic brain injury (TBI) is a major health problem that often requires intensive and long-term rehabilitation.

**Objective:**

The aim of this study was to determine whether rehabilitative digital gaming facilitates cognitive functioning and general well-being in people with TBI.

**Methods:**

A total of 90 Finnish-speaking adults with TBI (18-65 years) were recruited from an outpatient neuroscience clinic. The participants were randomly allocated to one of the three groups: a rehabilitation gaming group (n=29, intervention), an entertainment gaming group (n=29, active control), or a passive control group (n=32). The gaming groups were instructed to engage in gaming for a minimum of 30 min per day for 8 weeks. Primary and secondary outcomes were measured at three time points: before the intervention, after the intervention, and 3 months following the intervention. The primary outcome was cognitive status measured by processing speed and visuomotor tasks (The Trail Making Test; Wechsler Adult Intelligence Scale-Fourth Edition, WAIS-IV, symbol search, coding, and cancellation tasks). Secondary outcomes were attention and executive functions (Simon task), working memory (WAIS-IV digit span and Paced Auditory Serial Addition Test, PASAT), depression (Patient Health Questionnaire-9), self-efficacy (General Self-efficacy Scale), and executive functions (Behavior Rating Inventory of Executive Function-Adult Version). Feasibility information was assessed (acceptability, measurement instruments filled, dropouts, adherence, usability, satisfaction, and possible future use). Cognitive measurements were conducted in face-to-face interviews by trained psychologists, and questionnaires were self-administered.

**Results:**

The effects of rehabilitation gaming did not significantly differ from the effects of entertainment gaming or being in a passive control group. For primary outcomes and PASAT tests, the participants in all three groups showed overall improvement in test scores across the three measurement points. However, depression scores increased significantly between baseline and after 8 weeks and between baseline and after 3 months in the rehabilitative gaming group. No differences were found in patients’ self-efficacy between the three measuring points in any of the groups. Participants did use the games (rehabilitation group: 93%, 27/29; entertainment group 100%, 29/29). Games were seen as a usable intervention (rehabilitation group: 70%, 14/29; entertainment group: 83%, 20/29). The rehabilitation group was less satisfied with the gaming intervention (68%, 13/29 vs 83%, 20/29), but they were more willing to use the game after the intervention period (76%, 16/29 vs 63%, 15/29). Total time spent on gaming during the intervention period was low (15.22 hour rehabilitation gaming group, 19.22 hour entertainment gaming group).

**Conclusions:**

We did not find differences between the groups in improvement in the outcome measures. The improvements in test performance by all three groups may reflect rehearsal effects. Entertainment gaming had elements that could be considered when rehabilitative games are designed for, implemented in, and assessed in larger clinical trials for persons with TBI.

**Trial Registration:**

ClinicalTrials.gov NCT02425527; https://clinicaltrials.gov/ct2/show/NCT02425527 (Archived by WebCite at http://www.webcitation.org/6esKI1uDH)

## Introduction

### Significance of Traumatic Brain Injury

Traumatic brain injury (TBI) is a major cause of long-term disabilities. In the United States, at least 5.3 million citizens live with disabilities resulting from TBI [1], and in Europe, there is an overall incidence rate of 262 per 100,000 people per year [2]. In Finland (a nation of 5.6 million inhabitants), over 20,000 people suffer from TBI each year [3], and around 100,000 live with disabilities resulting from TBI [4]. Besides emotional symptoms and fatigue [4], cognitive deficits in attention and short-term memory are among the most common and disabling characteristics of people with TBI [5]. The recovery process requires complex, intensive, and long-term–assisted rehabilitation programs [6-8] that inflict a great burden on affected individuals [9] and also on health systems [10]. Individuals with brain injury are suggested to benefit from early and long-term therapeutic interventions [11], and the course of treatment for brain injuries is supported by clinical care guidelines [4,12,13]. Coherent evidence to support the effectiveness of interventions is still scarce [14].

Previous studies have found that rehabilitation interventions after TBI have increased participants’ attention, memory, social communication skills, and executive functions [15]. Carney et al [16] concluded in their systematic review, based on two randomized controlled trials (RCTs) and one observational study, that specific forms of cognitive rehabilitation reduce memory failures and anxiety and improve self-concept and interpersonal relationships for persons with TBI. Metacognitive strategy training focusing on functional everyday activities has also been proposed as an appropriate method for rehabilitating people with TBI [17].

### Gaming in Improving Functioning of People With Traumatic Brain Injury

A systematic review by Spreij et al [18] has further suggested that computer-based cognitive retraining is one of the most promising novel approaches in improving memory function after an acquired brain injury, although the results are currently inconclusive [19]. Until recently, a majority of patients (75%) with TBI have been younger than 35 years [20]. Likewise, 30% of video game players are in the age range of 18 to 35 years [21], and an average young person has played a total of 10,000 hours of video games by the age of 21 years [22]. It can therefore be assumed that gaming could be a feasible and engaging method in cognitive rehabilitation, especially for young people with TBI. This is supported by the systematic review by Primack et al [23], who concluded that video games have the potential to improve health outcomes in psychological and physical therapy [23]. Gaming has already been used in rehabilitation among people with multiple sclerosis [24], rheumatoid arthritis [25], diabetes [26], complex chronic pain and fatigue [27], spinal cord injury [28], and stroke patients [29,30]. A meta-analytic study of 21 experimental studies by Toril et al [31] indicates that video game training produces positive effects on cognitive functions, including reaction time, attention, memory, and global cognition, although because of the high heterogeneity of the studies, the results must be interpreted with caution. Action video game players have also shown better performance in alertness and cognition compared with those who do not play games [32,33].

Kühn et al [34] found that, for healthy adults, gaming significantly increased gray matter in the right hippocampal formation, right dorsolateral prefrontal cortex, and bilaterally in the cerebellum. The authors concluded that gaming can improve several cognitive functions. Lampit and colleagues [35] reported that computerized cognitive training in elderly healthy adults was modestly effective in improving cognitive performance. However, efficacy varied across cognitive domains and was largely determined by design choices. Bavelier et al [36] have also shown that playing action video games produces significant improvements in attentional control in healthy adults [36]. Furthermore, Ball and colleagues [37] conducted a large-scale cognitive training study and found that, although there was no transfer to other untrained skills, training improved memory, attention, and problem-solving skills. On the other hand, it has been suggested that gaming programs are inadequate for efficient integration in current clinical practice [38,39].

Gaming has already been used to some extent in the rehabilitation of persons with TBI [40]. Vakili et al [41] conducted a controlled study on the effects of video games in the rehabilitation of TBI patients. A total of 31 male TBI patients in the age range of 18 and 65 years were allocated to either a treatment group or a waitlist (treatment-as-usual) control group. The treatment group attended a 2-hour group rehabilitation session once a week for 8 weeks. During these sessions, about one-quarter of the time was dedicated to psychoeducation and the rest of the time for playing the action video game Medal of Honor: Rising Sun. The treatment group’s attentional performance improved in several behavioral measures (namely the Attentional Blink task and some subtasks of the Test of Everyday Attention), as did their self-reported quality of life (QoL; measured with the Comprehensive Quality of Life Scale-Fifth Edition). However, gaming did not have a significant effect on self-reported executive control (as measured by the Behavior Rating Inventory of Executive Functioning-Adult version, BRIEF-A) or self-efficacy (as measured by the General Self-Efficacy Scale, GSE). Although this study with a rather small sample size does showcase gaming as a noteworthy candidate for rehabilitation of TBI, the effect of the psychoeducation part of the treatment is not controlled for.

The scientific evidence for the effectiveness of gaming for enhancing cognitive functioning is mixed at best, and more studies in this area are needed [42,43]. Targeting persons with brain injury is relevant because it is still unknown whether the benefits of video game training can be transferred to clinical settings [34] or whether games can improve cognitive functions important for the management of daily activities [44]. It would also be prudent to explore whether gaming has any positive effects for TBI patients within a broader age range. Previous studies concerning the effects of video gaming on cognition have mostly been conducted with young people [45]. It has also been shown that participants in the age range of 6 and 29 years with acquired brain injury have exhibited significant improvement in processing speed, visual-motor coordination, and response inhibition after playing sessions over 12 weeks with two, 1-hour-long training periods with Nintendo Wii [46]. As TBI often results in long-term disability with adverse social, psychological, and economic consequences, it is important to seek methods that optimize independence and social participation to reduce long-term care needs and enhance QoL [47] for adults with TBI.

In this study, we aim to evaluate the effects and feasibility of digital games for improving cognitive functioning and well-being among people with TBI. We hypothesized that among patients with TBI in the intervention group (rehabilitation gaming), in comparison to the active control group (entertainment gaming) and passive control group, there would be a greater improvement in cognitive functioning (processing speed and visuomotor tasks, attention and executive functions, and working memory) and well-being (depression and self-efficacy).

## Methods

### Trial Registration

The trial has been registered in trial register ClinicalTrials.gov (NCT02425527).

### Design

The study includes a three-arm, parallel, and randomized clinical trial examining the effectiveness and feasibility of digital gaming for improving cognitive functioning and general well-being in people with TBI. The full study design and detailed description of the study methods can be found elsewhere [48].

### Sample Size

On the basis of our preliminary power calculations (see [48]), the sample size was expected to be 30 in each group, which is not very strong but reasonable enough for a feasibility study aiming to detect changes within a group between baseline and follow-up outcome measurements with an expected attrition rate close to 0%. However, some patients changed their minds regarding their participation before signing a consent form and dropped out of the recruitment process. Therefore, we needed to recruit more patients (n=106) to have 90 participants for randomization.

### Participants and Inclusion and Exclusion Criteria

The study was conducted at the Turku University Hospital, Division of Clinical Neurosciences in Turku, Finland.

The eligibility criteria stipulated that participants must be Finnish-speaking and reading adults, in the age range of 18 and 65 years old, and who have been diagnosed with TBI (ICD-10, S06.X, T90.5). To avoid any confounding factors, they should not have had active participation in cognitive rehabilitation (remediation therapy) during the 3 months before the intervention. To ensure that the participants were comparable regarding their clinical status and able to manage their intervention in their home environment, they should have been discharged from the hospital at least 12 months before the recruitment. In addition, eligible participants had to own a TV and a computer and have Internet access at home.

To see possible effects of the gaming, active digital gamers were excluded, with the allowed gaming time being 5 hours or less per week [36]. Potential participants were also excluded if they had sensory impairment (eg, serious visual impairment), severe cognitive impairment (eg, memory problems, slow processing speed, lack of attention, and linguistic problems), a physical impairment that may restrict the use of computers or computer game control systems unaided (eg, hemiplegia and dysfunction of the central vestibular system), apathy identified in previous neuropsychological evaluations, or a diagnosis of a severe mental disorder (eg, schizophrenia or severe depressive disorders identified as the secondary diagnosis). Patient eligibility was assessed primarily by reviewing Turku University Hospital electronic medical records, after which potentially eligible patients were further interviewed via telephone and assessed face-to-face by a trained psychologist.

### Recruitment

The hospital electronic medical records were accessed (June 2015), and patients with a TBI diagnosis were screened to determine which patients fulfilled the eligibility criteria for study participation. Those patients who were assessed to meet the inclusion criteria were contacted by telephone or by mail from June 22, 2015 to November 24, 2015 by researchers. Eligible participants with preliminary interest toward the study received written information about the study by mail in addition to informed consent forms, baseline questionnaires to be filled out, and a short description of the eight entertainment games (in case of allocation to the entertainment gaming group). They were contacted again after 1 to 2 weeks by telephone to enquire whether they would like to participate in the study and what type of game they would like to play if they were allocated to the group of entertainment gaming. If the contacted individual was interested in participating, the trial manager then received a message (by email, SMS text message [short message service, SMS], or telephone) sent by the recruiting researcher and allocated the patient randomly to one of the three arms of the trial. Eligible participants were then invited to the research laboratory, at which time an informed consent form was signed.

The participants’ baseline data were gathered, and cognitive measurements were conducted by a trained psychologist at the test laboratory. At the end of the study, three gaming consoles used in the study were given to randomly chosen study participants.

### Randomization and Masking

The study was individually randomized. The randomization and patient allocation were fully centralized (at the University of Turku). An independent trial statistician outside the study group randomly assigned (a block randomization in three blocks) the participants using randomization software (SAS [SAS Institute Inc] for Windows, version 9.3). The randomization list was delivered to the trial manager outside the study group. The trial manager informed the researchers about participants’ group after the baseline assessments. The researchers overseeing patient recruitment and randomization were therefore aware of the assignments. Due to the intervention type, allocation was not masked to participants in the intervention and control groups or to researchers who recruited patients. The psychologists, as cognitive outcome assessors, were kept blinded. However, in some occasions, study participants told them about their possible game playing. The data analyst (the trial statistician) was kept blinded to the allocation. As far as we are aware, there was no contact between participants in different groups, as they lived throughout a wide geographic area inside the university hospital catchment area.

### Interventions

#### Rehabilitation Gaming

Patients in the rehabilitation gaming group (intervention group) used an Internet browser–based digital brain training program, CogniFit [49]. We used a Web-based cognitive training platform with 33 games designed with the purpose of improving the user's cognitive abilities as brain exercises. To ensure a user-centered approach, the participants were instructed to play at least one exercise from each of the three categories (memory, spatial perception, and mental planning) during each training session daily, otherwise, they were free to choose which exercises they wished to play. Giving participants a sense of agency aimed to increase the likelihood that participants engaged in gaming as instructed [50].

To support the participants’ gaming activities and fidelity for the gaming, written instructions for the rehabilitation game were given to the participant. In addition, instructions for the rehabilitation game were introduced to the participants during the introductory meeting with the researcher (two different researchers, both registered nurses and masters’ degree in nursing science), which took about 30 min per person. During the meeting, participants’ abilities and previous experience in playing digital games were explored to ensure that the participants had the basic gaming skills required for active gaming. A new email address, a password for the email account, and a personal game account were generated for each participant, as the browser-based program required access through a website, and the user would log in with an email address and a specified password. The participant also tested the game unaided to find out possible barriers in their gaming. To record participants' progression and scores on each of the games, the research team had access to the program, and the progress of each participant’s game score was monitored. The participant had also a possibility to monitor their own progress in the program. Information about the frequency of training sessions was also recorded by the participants themselves in a gaming diary. The participants’ adherence to and motivation [51] for gaming were supported and monitored by weekly telephone calls. During these telephone calls, participants had also the possibility report any technical problems. The telephone calls were made by two researchers and one research assistant (RA). Researchers had qualifications of registered nurse and masters’ degree in nursing science, and the RA had a degree of public health nurse and bachelor’s degree in nursing science.

The participants were guided to use the rehabilitation game for at least 30 min per day [34,52,53] over a period of 8 weeks. To encourage, motivate, and hold participants to training, they were supported in planning a schedule for their training sessions (days, time, and frequency) for the entire 8-week gaming period. A more detailed description of the intervention can be found in the study protocol [48].

#### Entertainment Gaming

Participants in the entertainment gaming group (active control group) used commercial digital games designed for Sony PlayStation 3 (PS3) consoles. The project purchased the participant-selected game (see below) from the official PlayStation Store and downloaded and installed the game into the console given to the participant. Games to be selected by the participants (a total of eight games) were considered to correspond to the rehabilitation games and to contain the same core gameplay elements (see [48]).

As in the intervention group, the participants chose an entertainment game that they found enjoyable, which was assumed to increase the likelihood that participants engage in gaming as instructed and to ensure the attractiveness of the game for the player. However, the participants were not forced to play any one type of game, and they were able to change the game during the 8-week intervention period if they had concerns, for example, because of violent content. Again, during the introductory meeting (about 30 min per person), written instructions regarding how to use the console were given to the participant, and the game the participant selected was tested with the researcher (same researchers as with the rehabilitation gaming group). As with the intervention group, ability to play digital games was explored to ensure that participants had the basic gaming skills required for active gaming. An overview of the use of the console was also offered, and a tutorial demonstration was given (how to start the console; how to play the game; how to use the controller; how to change game options, such as game difficulty and speed; and so on). A technical assistant was available to visit the participant’s home to help set up the console [54] or give guidance by telephone. The participants were guided to play the console for at least 30 min per day over a period of 8 weeks [34]. The participants were supported in planning their training session schedule (days and times), and information about game sessions (day, time, frequency, and play progress) was recorded by the participant in a gaming diary. Therefore, a participant was also able to monitor his or her own progress in the game. Furthermore, adherence to gaming was supported and monitored by weekly telephone calls. As in the intervention group, during these telephone calls, participants had the possibility report any technical problems. The telephone calls were made by the same researchers and RA than in the rehabilitation gaming group.

### No Gaming

Patients in the no-gaming group (passive control group) did not have gaming activities organized by the project, but as with the gaming groups, the researchers called them weekly. Participants in this group were offered an opportunity to have games and consoles for a 2-week period free of charge after the study; 11 did so after the follow-up measurement.

### Assessment

Patient data were collected at three different times: at baseline, after the intervention (8 weeks, from September 2015 to December 2015), and 3 months after the intervention ended (from December 2015 to April 2016). Cognitive tests were conducted in the research laboratory, and self-administered questionnaires were sent to participants homes to be filled out before each visit to the research laboratory. Participants returned the questionnaires during the visit to the research laboratory.

### Outcomes

#### Primary Outcome

##### Processing Speed and Visuomotor Tasks

The Trail Making Test (TMT) requires visual search, scanning, speed of processing, mental flexibility, and executive functions [55]. The test consists of two parts, A and B. In TMT A, participants are given a paper displaying circles numbered 1 to 25 in random order; the task is to draw lines that will connect the numbers in ascending order. In TMT B, the circles contain both numbers and letters. In this part of the test, the task is to draw lines to connect the circles so that they alter between numbers and letters in an ascending order (1-A-2-B and so on). The time it takes to complete the trail in each part is recorded (see [48]). TMT and the WAIS-IV subtests are recommended outcome measures in TBI research because of their reliability and validity [56].

In addition to the TMT, three tasks from the WAIS-IV test package were used to further assess processing speed and visuomotor skills: (1) symbol search, (2) cancellation, and (3) coding. The three tasks chosen for this experiment are aimed toward assessing skills of sorting out simple visual information, monitoring, making progress in a task, maintaining attention, visuomotor coordination, and visual memory [57]. In both the symbol search and the cancellation tasks, the participants perform a visual search to find out if a certain symbol is among other symbols. In the symbol search task, the symbols are organized in rows, and the participant must indicate for each row whether or not a required symbol appears on the row. The cancellation task is similar to the symbol search task, but this time the participant seeks to find set symbols during the whole task (instead of the required symbols changing on each row). In the coding task, the participant is given a set of number-symbol pairs. The task is to fill out an empty grid containing only numbers with the appropriate symbols matching those numbers (see [48]).

#### Secondary Outcomes

##### Attention and Executive Functions

The Simon task [58,59] was used to measure the inhibition component of executive functions [60]. In the task, a blue or red square appears on either the left or right side of the screen. The participant is instructed to push the left button on a response pad each time a blue square appears and the right button each time a red square appears, irrespective of which side the square is presented. In congruent trials, the response button is on the same side as the square, and in incongruent trials the square is on the opposite side of the response button (ie, the irrelevant spatial information is conflicting with the correct response). The number of correct responses and reaction times are recorded, and the difference between the congruent and incongruent trials is used as a measure of the Simon effect (see [48]).

##### Working Memory

Working memory was assessed with the digit span task from the WAIS-IV package [57]. In the first part of the task, participants repeat numbers in the order they heard them. In the second part, they repeat the numbers backwards. In the third part of the task, the participants repeat numbers in numerical order [57]. WAIS-IV subtests have been recommended as outcome measures in TBI research because of their reliability and validity [56].

The Paced Auditory Serial Addition Test (PASAT) [61] measures auditory information processing speed, flexibility, and calculation skills [62]. There are two parts in this task. In the first part, single numbers are presented every 3 seconds. The participant adds each new number to the last number before it. In the second part, the numbers are presented every 2 seconds. The test score is the number of correct sums given in each trial (see [48]). PASAT is widely used to assess cognitive changes in TBI patients, and it has good psychometric properties, even though some studies suggest that it may be sensitive to practice effects [63].

##### Depression

In the Patient Health Questionnaire-9 (PHQ-9) [64,65], a self-administered questionnaire, respondents are asked to indicate how often they have been bothered by any of the problems over the previous 2 weeks, such as little interest or pleasure in doing things, feeling down, depressed, or hopeless, feeling tired or having little energy. Each of the nine items are scored as 0=not at all, 1=several days, 2=more than half of the days, and 3=nearly every day. On the basis of the individual items, a total score is formed; the higher the score, the more severe the depression symptoms (range: 0-27). The measure has demonstrated diagnostic sensitivity and strong reliability in previous studies among TBI populations [66,67].

##### Self-Efficacy

The GSE [65] is a self-administered scale that assesses a general sense of perceived self-efficacy to predict coping with daily challenges, as well as adaptation after experiencing a variety of stressful life events. The scale consists of 10 items, and responses are made on a 4-point scale (1=not at all true, 2=hardly true, 3=moderately true, and 4=exactly true). It takes about 4 min to complete. The final composite score ranges from 10 to 40 and comprises the sum of all 10 responses; low scores represent a lower ability to cope with daily problems. The scale has been previously used in studies with TBI populations [68,69].

##### Executive Functions

The BRIEF-A is a 75-item self-administrated questionnaire that focuses on executive functions in daily life [70]. Responses are given in a 3-point Likert scale (never or sometimes or often), and a global executive composite score is formed by the total score [70] (see [48]). BRIEF-A has been shown to have good psychometric qualities in a sample of TBI patients [71].

##### Feasibility

Cumulative monitoring was conducted during the 8-week period regarding gaming activities (gaming frequency, timing, and time) in both gaming groups (intervention and active control group). The gaming information concerning the rehabilitation gaming group were collected from game logs retrieved from the gaming system. Regarding entertainment games, the information was collected from console gaming logs where possible and from the gaming diaries where the logs were not available. Feasibility was assessed by collecting the following information during the study process: acceptability as measurement instruments filled out (yes, no; %), attrition as calculating dropouts for any reason (yes, no; %), and adherence as involvement in the interventions for an 8-week period (yes, no; %). Feasibility evaluation in terms of usability, satisfaction, and future use was assessed by asking the participants: Was the game usable? (yes or no; %), Have you been satisfied with the game? (yes or no; %), and Would you like to use the game in the future? (yes or no or maybe; %). The participants had a possibility to specify their answers by answering to open-ended questions (not analyzed in the study because of limited size of the data). In addition, participants’ selections of the commercial digital games designed for Sony PS3 consoles are presented.

##### Background Information

Background information including sociodemographic characteristics and medical history was collected (age, gender, marital status, level of education, employment status, living situation, illness history, and current digital game playing [hours a week]).

### Statistical Methods

The sample size needed for the study was based on preliminary estimations (see [48]). The primary and secondary outcomes were assessed at baseline, after the end of the intervention, and 3 months after the intervention ended (at 6 months from baseline). To test the study’s hypothesis, the data were analyzed with analysis of covariance, in which Group (rehabilitation gaming, entertainment gaming, and passive control) was a between-subjects factor, and Time (before intervention, after intervention, and 6 months after baseline) was a within-subjects factor. Age was used as a covariate. Effectiveness of the intervention was indicated by a significant Group*Time interaction, which indicated differences between the three groups in the improvement of the primary and secondary outcomes over time.

For sensitivity analysis, we performed analyses for completer-only data and imputer data. We compared the study results between these two groups. No differences between the results were found in the intention to treat analysis or among those completing the follow-ups.

Statistical analyses were performed with SAS system for Windows, version 9.4 and Statistical Package for the Social Sciences (SPSS) statistics version 22 (IBM Corp). *P* values less than .05 are considered statistically significant.

### Ethical Issues

The study was evaluated by the Ethics Committee of the Turku University Hospital (ETMK 41/1801/2015), and the permission to conduct the study was granted by Turku University Hospital (T89/T04/008/2015). The trial has been officially registered (NCT02425527). All participants volunteered for the study. The study participants were informed orally (at least two telephone calls and one face-to-face meeting) and in written format of how and where their information was to be accessed, what the purpose of the study was, and what specific steps to be taken were to be (if agreed to participate in the study). Written informed consent was obtained in accordance with the Declaration of Helsinki [72]. To identify any ethical or practical concerns in the study protocol, entertainment and rehabilitation games were pretested with five healthy adults and with five people with TBI. On the basis of pretests, more specific inclusion and exclusion criteria for the study were identified. In addition, some games initially identified to be used in the study were excluded if they were suspected to cause dizziness or headaches because of dark colors or three-dimensional tunnel effects [73].

## Results

### Sample Characteristics

**Figure 1:**
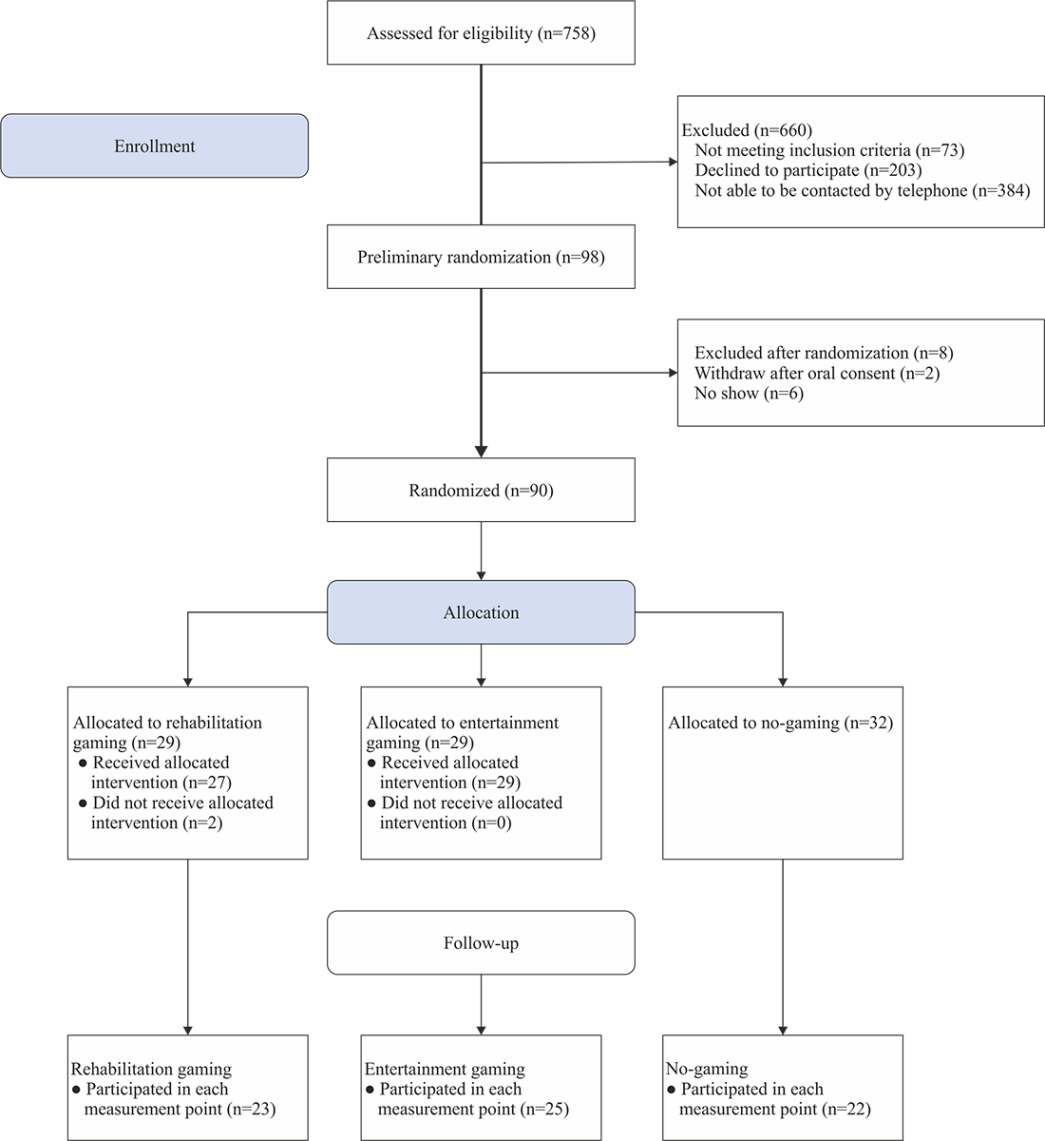
Flowchart.

The flowchart of the participants is described in Figure 1. A total of 758 individuals were screened for eligibility to participate. Of these, 660 were excluded from the study (73 did not meet the inclusion criteria, 203 refused to participate, and 384 could not be contacted by telephone [no answer after two attempts, incorrect or lack of telephone number]). A total of 8 people withdrew their consent: two people refused before randomization, and 6 patients did not show up for the first research meeting. Out of 106 people recruited, 90 people were randomly allocated to the intervention group (n=29), the active control group (n=29), or the passive control group (n=32). At the follow-up, the response rate for the intervention group was 79%, 86% for the active control group, and 69% for the passive control group (the attrition rate was 21%, 14%, and 31%, respectively).

The mean age of all participants was 41 years, and half (45/90, 50%) were male. Over half of the participants were married (53/90, 59%) or lived with a partner (54/90, 60%). More detailed characteristics of the participants are described in Table 1.

Out of all participants, 43% (39/90) had played digital games weekly before the trial: the highest previous gaming activity was in the rehabilitation gaming group (48%, 14/90), followed by the entertainment gaming group (45%, 13/90), and the passive control group (34%, 11/90). Over half of the participants (57%, 51/90) had not previously played any games (the passive control group: 66%, 21/90; the rehabilitation gaming group: 52%, 15/90; and the entertainment gaming group: 55%, 16/90).

Description of the outcome information at baseline is described in Table 2. Comparisons of the groups at baseline showed no evidence of differences between the groups in any of the measures.

**Table 1:** Background characteristic of the participants.

Characteristic		Rehabilitation gaming (N=29)	Entertainment gaming (N=29)	No-intervention (N=32)
Age (years), mean (SD)		42.14 (12.15)	40.90 (12.01)	39.34 (12.08)
**Sex, n (%)**				
	Male	15 (52)	13 (45)	17 (53)
**Marital status, n (%)**				
	Single	10 (35)	7 (24)	5 (15)
	Married	15 (52)	17 (59)	21 (66)
	Divorced	3 (10)	4 (14)	6 (19)
	Widowed	0 (0)	1 (3)	0 (0)
**Living situation, n (%)**				
	Alone	10 (35)	11 (38)	9 (28)
	With partner	17 (59)	16 (55)	21 (66)
	Other	1 (3)	2 (7)	2 (6)
**Level of education, n (%)**				
	No formal education	0 (0)	0 (0)	1 (3)
	Secondary grammar school	3 (11)	3 (10)	1 (3)
	High school	1 (4)	3 (10)	5 (16)
	Vocational education	13 (46)	17 (59)	12 (38)
	University degree	11 (40)	6 (21)	12 (37)
	Doctoral degree	0 (0)	0 (0)	1 (3)
**Employment status, n (%)**				
	Employed	9 (32)	12 (41)	15 (47)
	Retired	10 (36)	10 (35)	8 (25)
	Student	3 (11)	1 (3)	5 (16)
	Job seeker	2 (7)	2 (7)	1 (3)
	Other	4 (14)	4 (14)	3 (9)
Duration of traumatic brain injury (months), mean (SD)		122 (133)	137 (107)	84 (101)

**Table 2:** Baseline information of different study groups.

Measure		CogniFit		PlayStation 3		Control		*F* (degrees of freedom) value	*P* value
		n	Mean (SD)	n	Mean (SD)	n	Mean (SD)		
TMT^a^ A		29	35.28 (15.84)	29	34.31 (12.61)	31	30.45 (12.37)	1.06 (2,86)	.35
TMT B		29	82.83 (54.08)	29	75.72 (24.42)	31	74.87 (28.57)	0.39 (2,86)	.68
WAIS-IV^b^ symbol search		29	33.00 (11.32)	29	33.00 (8.21)	31	34.84 (7.71)	0.41 (2,86)	.67
WAIS-IV symbol coding		29	65.00 (21.51)	29	63.79 (12.84)	31	66.84 (16.24)	0.24 (2,86)	.79
WAIS-IV symbol cancellation		29	38.31 (12.40)	29	37.83 (8.06)	31	39.06 (10.39)	0.11 (2,86)	.90
Attention and executive function (Simon test)		29	41.53 (39.94)	28	55.20 (45.64)	31	40.03 (36.76)	1.21 (2,85)	.30
**Working memory**									
	WAIS-IV digital span	29	25.79 (7.07)	29	26.03 (5.23)	31	26.32 (4.69)	0.06 (2,86)	.94
	PASAT^c^ 3 s	28	43.89 (14.20)	26	44.42 (10.40)	30	46.07 (10.45)	0.27 (2,81)	.77
	PASAT 2 s	27	35.59 (10.70)	26	32.38 (8.37)	29	35.48 (9.94)	0.93 (2,79)	.40
Depression (PHQ-9^d^)		29	5.79 (3.92)	29	4.66 (5.20)	32	6.34 (6.28)	0.81 (2,87)	.45
Self-efficacy (GSE^e^)		29	29.86 (5.55)	29	29.55 (4.77)	32	30.00 (4.87)	0.06 (2,87)	.94
Executive functions (BRIEF-A^f^)		20	109.25 (22.02)	20	114.60 (30.75)	21	121.57 (29.40)	1.02 (2,58)	.37

^a^TMT: Trail Making Test.

^b^WAIS-IV: Wechsler Adult Intelligence Scale-Fourth Edition.

^c^PASAT: Paced Auditory Serial Addition Test.

^d^PHQ-9: Patient Health Questionnaire-9.

^e^GSE: General Self-Efficacy Scale.

^f^BRIEF-A: Behavior Rating Inventory of Executive Functioning-Adult version.

### Effects of the Intervention

The analysis of the WAIS-IV *symbol search* scores showed no indication of a Group x Time interaction (*F*_4,138_=0.34, *P*=.85), which means that we failed to observe differences among groups in test improvement. However, there was a main effect of Time (*F*_2,138_=4.62, *P*=.01), reflecting overall improvement in test scores across the three measurement points. The main effect age (*F*_1,69_=5.68, *P*=.02) indicated that older participants had overall lower scores. Furthermore, regarding the WAIS-IV *coding* task, there was no indication of a Group x Time interaction (*F*_4,140_=1.11, *P*=.35). However, there was a main effect of Time, (*F*_2,140_=6.29, *P*=.002), indicating overall improvement in the test scores over time. Main effect of age indicated that older participants received lower scores (*F*_1,70_=6.99, *P*=.01). In the WAIS-IV *cancellation* task scores, there was no evidence for a Group x Time interaction (*F*_4,140_=0.69, *P*=.60). The only statistically significant effect was the main effect of age (*F*_1,70_=5.92, *P*=.02), indicating that older participants had lower scores. Thus, although there was overall improvement in the symbol search and coding tasks over time, no differences between groups in the magnitude of the improvement were indicated (see Multimedia Appendix 1).

The results of the TMT version A showed no indication of a two-way interaction between Group and Time (*F*_4,140_=0.51, *P*=.73), indicating that we failed to observe differences among groups in test improvement in this task. The analysis only indicated a main effect of Age (*F*_1,70_=11.17, *P*=.001), reflecting overall lower test scores for older participants. In the results of TMT version B, there was no indication of a Group x Time interaction (*F*_3.51,122.77_=0.31, *P*=.85) or other effects (see Multimedia Appendix 1).

#### Secondary Outcomes

##### Attention, Executive Functions and Working Memory

The *Simon task* results revealed no indication of an interaction between Time and Group, (*F*_3.32,114.44_=0.16, *P*=.94; see Multimedia Appendix 2). There was a main effect of age (*F*_1,69_=8.76, *P*=.004), indicating that the Simon effect (ie, the difference in the reaction time between congruent and incongruent trials) was greater for older participants.

In the PASAT 3 s version, there was no indication of an interaction between Group and Time (*F*_3.51,108.93_=1.20, *P*=.33) nor other effects. In the results of the PASAT 2 s version, there was no indication of an interaction (*F*_3.55,110.17_=0.57, *P*=.67), but there was a main effect of Time (*F*_1.78,110.17_=9.23, *P*<.001). In other words, we observed overall improvement in the test performance across the three measurement points (see Multimedia Appendix 2).

For the WAIS-IV *digit span* task, there was no indication of an interaction (*F*_4,138_=0.80, *P*=.53), nor of any main effects (see Multimedia Appendix 2).

##### Depression

The results for the depression score outcomes are described in Multimedia Appendix 2. The depression score results revealed no indication of an interaction between Time and Group, (*F*_4,134_=0.848, *P*=.48). There was no main effect of Time (*F*_2,134_=2.212, *P*=.11). The analysis showed that depressive symptoms remained nearly the same between baseline and 3 months, both in the entertainment group (mean 4.12 [SD 4.94] to mean 4.28 [SD 4.67], *P*=.51) and the passive control group (mean 6.36 [SD 5.91] to mean 6.46 [SD 6.35], *P*=.84). On the contrary, in the rehabilitation gaming group, the mean scores increased from baseline to 8 weeks and from baseline to 3 months (mean 5.04 [SD 3.82] to mean 6.65 [SD 5.00], *P*=.05), showing increase in the participants’ depressive symptoms. On the categorical level (mild vs moderate depression), the change observed between time points was not clinically significant (scoring 6-9 points indicates minimal symptoms, University of Michigan Health System (UMHS) Depression Guideline, August 2011).

##### Self-Efficacy

The general self-efficacy scores results revealed no indication of an interaction between Time and Group, (*F*_3.77,126.19_=0.534, *P*=.70). There was no main effect of Time (*F*_1.88,129.19_=1.38, *P*=.26). Self-efficacy among the participants increased slightly in the passive control group (*P*=.06) over time, but the change was not statistically significant (see Multimedia Appendix 2).

##### Executive Functions

The analysis of the BRIEF-A scores revealed no indication of an interaction between Time and Group, (*F*_4,114_=1.99, *P*=.10). The main effect of Time (*F*_2,114_=3.54, *P*=.03) indicated that there were differences between measurement points (see Multimedia Appendix 2). A post hoc comparison between baseline and 8 weeks after the intervention was conducted. No statistically significant differences between these two time points were found.

##### Feasibility

**Figure 2:**
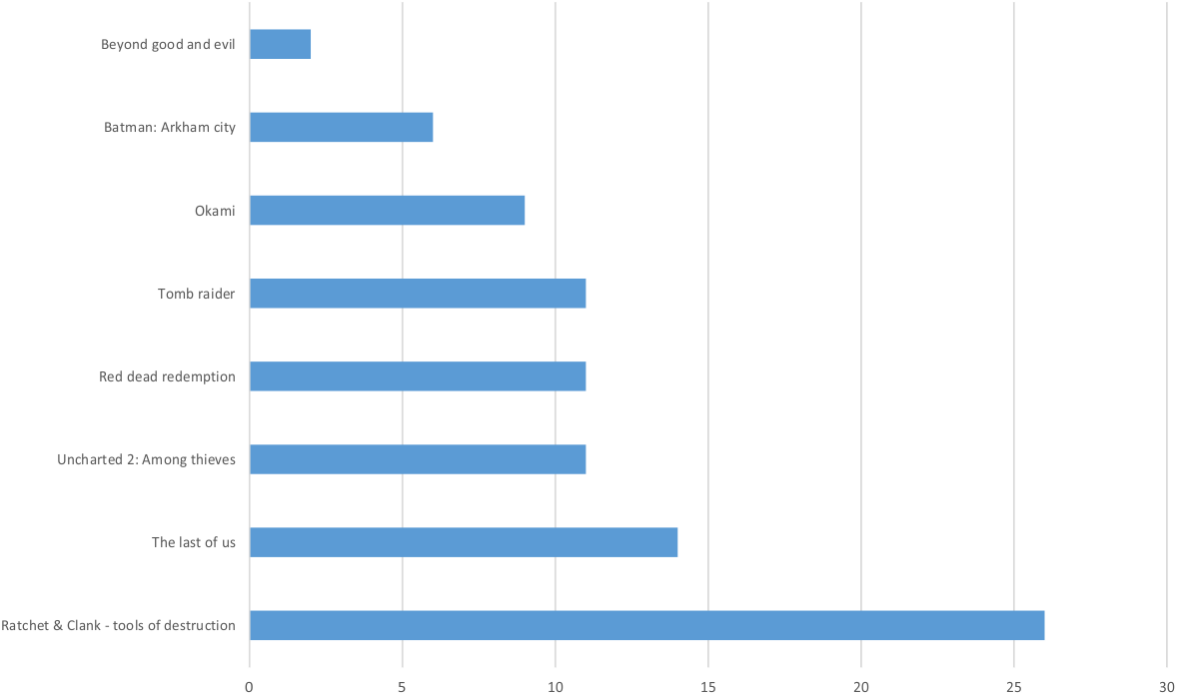
Selections of entertainment games by participants (each participant could change their game and therefore select more than one game).

The most favorable game was Ratchet and Clank—Tools of Destruction (26/90) and the Last of us (14/90). The least favorable games were Beyond Good and Evil and Batman: Arkham City (Figure 2).

Out of 758 patients screened, the refusal rate was 27%. A total of 20 randomized participants dropped out of the study during the intervention period (attrition rate 21% (6/29) in the rehabilitation group, 14% (4/29) in the entertainment group, and 31% (10/32) in the passive control group; Table 3).

**Figure 3:**
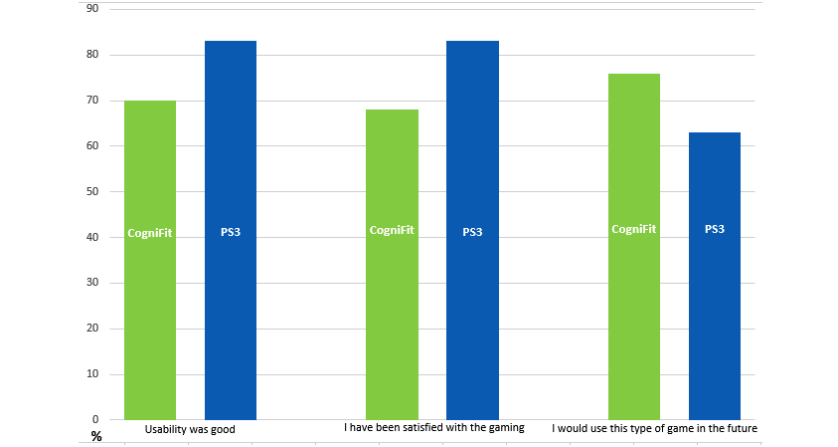
Comparison of the usability, satisfaction, and future use between rehabilitation gaming and entertainment gaming groups.

During the 8-week intervention period, the average gaming time in the entertainment gaming group was 19.22 hours (range 0-71.48 hours) and in the rehabilitation gaming group 15.02 hours (range 0.12-71.38 hours). In general, the participants were adherent to the intervention (entertainment group 100%, 29/29, rehabilitation group 93%, 27/29), and they attended the prescheduled testing sessions (86%, 25/29, in the entertainment group, 79%, 23/29, in the rehabilitation group). Most participants in the entertainment group (83%, 20/29) and in the rehabilitation group (70%, 14/29) also agreed that the usability of the gaming was good, and about two-thirds (68%, 13/29) of the rehabilitation group and 83% (20/29) of the entertainment group were satisfied with the game. Contrary to our expectations, more participants in the rehabilitation group than the entertainment group were willing to use the type of game they were assigned after the intervention was finished as part of their rehabilitation process (76%, 16/29, vs 63%, 15/29; Figure 3).

**Table 3:** Criteria for the feasibility of rehabilitation and entertainment gaming.

Criterion		Rehabilitation gaming group (n=29), n (%)	Entertainment gaming group (n=29), n (%)
**Adherence**			
	Prescheduled measurements performed	23 (79)	25 (86)
	Participants’ dropout because of any reason	6 (21)	4 (14)
	The acceptability of the game	28 (95)	(29) 100
**Usability**			
	Usability evaluation for the gaming system (<80%)	14 (70)	20 (83)
**Satisfaction**			
	Satisfied with the games (<80%)	13 (68)	20 (83)
**Use in the future**			
	Willing to use the games later as part of their recovery process (<60%)	16 (76)	15 (63)

## Discussion

### Principal Findings

We evaluate the effects and feasibility of digital games for improving cognitive functioning and well-being among people with TBI. We found no differences between the control group and the two intervention groups for the primary outcomes (processing speed and visuomotor tasks) or any of the secondary outcomes. Test scores improved in all groups over time regarding several different variables.

As this improvement is not related to gaming (ie, whether the participants belonged to one of the two gaming groups or the control group), this effect was likely due to practice effects on the tasks used for assessing the outcomes, which compromises attempts to detect between-group differences in the improvement over time [74]. Indeed, the improvement in the postgaming performance was limited to the cognitive tasks and was not observed in, for example, depression symptoms. However, it is worth noting that in the rehabilitation group, the respondents’ depressive symptoms increased during the study period. This finding is important when a new intervention is introduced to the participants. We must, therefore, question whether gaming is associated with this increase of depressive symptoms, even though there is evidence of an association between depression symptoms or mood disorders and TBI [75-77]. Thus, in the future, studies should focus on how gaming might affect persons with TBI, to avoid any harm in patients’ clinical status and their QoL.

The age range in our study participants was broad (18-65 years), and our sample size was relatively small. Our study results would have been different if we would have narrowed the age of participants and aimed for a younger target population. This could be reasonable adjustment, keeping in mind that the majority of patients (75%) with TBI are younger than 35 years [20]. A systematic review of Shams et al [45] also showed that most studies aiming to improve cognitive functioning have targeted younger participants. On the other hand, we aimed to capture real-life events of individuals from a variety of age groups, who were not active gamers. We also wanted to facilitate gaming at home to increase participants’ engagement in intervention [78]. However, this decision may have caused another concern. We found low fidelity in the intervention, which may be a result of participants’ independent gaming intervention at home. Patients in the study by Lampit and colleagues [35] also found that training in an unsupervised home environment is not as effective as supervised training. On the other hand, the differences between the rehabilitation and entertainment game groups in adherence to the intervention and the experienced usability and satisfaction with the games could in part be related to differences between the samples: the rehabilitation group subjects were slightly more often single and less often employed than entertainment game group subjects. These factors may be related to depression, which also slightly increased during the study period in the rehabilitation game group. We observed a high attrition rate, especially in the passive control group, that is, 31%. This has also been found in earlier studies related to cognitive rehabilitation and TBI, such as the study by Vakili & Langdon (2016) with high attrition rate among control group [41]. Dropout in previous studies has been caused by fatigue [79], mental fatigue, or headache during computerized rehabilitation [80]. In our study, the reasons for dropout have not been systematically collected. On the basis of previous studies, to avoid loss of motivation in long training schedules, shorter gaming interventions (1-6 weeks) might be more beneficial for older adults [31]. The training may be seen as exciting at first, but may later be considered boring [81]. Furthermore, we are unaware of how many participants in the control group were engaged in gaming activities, a factor that could positively affect their cognitive status. Of course, there is the possibility that gaming simply is not effective in the rehabilitation of TBI. In addition, because of system updates in the intervention group, some technical errors appeared, and some functions in participants’ rehabilitation game user accounts changed (eg, prespecified gaming categories in CogniFit were not included in the participants’ user accounts). These changes affected 13 participants for 11 days, and it is therefore unlikely that this affected the results. Furthermore, even though we did include sensitive reaction time measurements among our secondary outcomes (ie, the Simon task), our primary measures were paper-and-pencil tests, which may not have been sensitive enough to capture subtle improvements in cognitive performance. In addition, the association of self-monitoring data concerning the participants’ gaming progress and the effects of the gaming could also be used to assess cofounding factors.

In the entertainment gaming group, the participants were given the chance to select their favorite game or change the game during the intervention. Although all the games included in this study were considered to contain similar game dynamics assumed to improve certain cognitive functions, there was some variability between the games, and it is possible that the game dynamics of the participant’s favorite game did not target the specific cognitive deficits of that participant. The choice of eight games also makes it difficult to conclude which types of game dynamics actually improve the cognitive functions of interest. In the future, a single game might be a better option in RCT design to ensure accuracy of the content of the different interventions.

The games used in this study may have also included too many action games, whereas participants might prefer other types of games. Vahlo et al [82] showed in their study that only about one-fifth of healthy adults enjoyed playing action-adventure games. As the dislike toward certain types of games or game activities can be rather strong, game selection should represent a wider variety of different game genres to meet personalized gaming preferences in future trials. Some incidental factors could also have shown to affect the outcomes of the results, such as patient perceptions or attitudes toward gaming. As far as we are aware, technological solutions are not used routinely in outpatient care for persons with TBI. If the participants do not see gaming as a seriously taken opportunity for rehabilitation, its effects may be questioned, and recommended gaming instructions may not be followed. We did not perform expectancy testing before the assigned intervention, which could be useful to avoid a placebo effect. We are not aware if patients’ prior expectancies of the effectiveness of the gaming affected the outcome of the study [83]. Therefore, the participants’ own perceptions toward gaming and its use as part of rehabilitative interventions should be explored in more detail. In the future, patients could potentially be prescribed personalized gaming interventions based on specific cognitive deficits and their personal game preferences, which would improve the effectiveness of the intervention.

Finally, the sample size of the study was small, making it difficult to detect small effects (ie, differences between groups), especially as the sample included a relatively heterogeneous group of patients with a wide variety of cognitive deficits. These factors limit the generalization of the results to a wider population. In future studies, a research design with a larger sample size is needed.

### Conclusions

To receive valid outcomes of the effectiveness of gaming, it would be important to make sure that the gaming dose is high enough. One way to do this is to ensure participants’ gaming is monitored daily.

## Appendix

Multimedia Appendix 1 Analysis of primary outcome, processing speed, and visuomotor task, on baseline, 8 weeks, and 3 months (analysis of covariance).

Multimedia Appendix 2 Analysis of secondary outcomes at baseline, 8 weeks, and 3 months (analysis of covariance).

Multimedia Appendix 3 CONSORT‐EHEALTH checklist (V 1.6.1).

## Abbreviations

BRIEF-A: Behavior Rating Inventory of Executive Function-Adult version
GSE: General Self-efficacy Scale
PASAT: Paced Auditory Serial Addition Test
PHQ-9: Patient Health Questionnaire
QoL: quality of life
RA: research assistant
RCT: randomized controlled trial
TBI: traumatic brain injury
TMT: Trail Making Test
WAIS-IV: Wechsler Adult Intelligence Scale, 4th Edition
