# Supplementary material for: Digital Gaming for Improving the Functioning of People With Traumatic Brain Injury: Randomized Clinical Feasibility Study
Source: J Med Internet Res. 2018 Mar 19;20(3):e77. doi: 10.2196/jmir.7618 (PMC5881042; doi:10.2196/jmir.7618)
Supplement: Multimedia Appendix 1 [file jmir_v20i3e77_app1.pdf]

Primary outcome	CogniFit				PlayStation 3				Control				Time x Group interaction
	N	Mean (SE)	95% CI	*P* value	N	Mean (SE)	95% CI	*P* value	N	Mean (SE)	95% CI	*P* value	*P* value
**TMT[a] A**				.67				.36				.14	.73
Baseline	23	32.53 (2.62)	27.31-37.75		27	33.60 (2.41)	28.79-38.40		24	31.78 (2.56)	26.68-36.88		
8 weeks	23	30.78 (2.94)	24.92-36.63		27	28.82 (2.70)	23.48-34.26		24	27.32 (2.87)	21.59-33.04		
3 months	23	27.65 (2.28)	23.11-32.20		27	27.04 (2.10)	22.85-31.22		24	26.13 (2.23)	21.68-30.57		
**TMT B**				.91				.06				.20	.85
Baseline	23	81.73 (7.95)	65.86-97.60		27	76.02 (7.32)	61.40-90.63		24	76.44 (7.78)	60.91-91.96		
8 weeks	23	69.65 (7.31)	55.06-84.25		27	65.04 (6.73)	51.60-78.48		24	59.06 (7.15)	44.79-73.33		
3 months	23	59.25 (5.39)	48.50-70.01		27	57.30 (4.96)	47.40-67.21		24	53.36 (5.27)	42.84-63.88		
**WAIS-IV[b] symbol search**				.47				.02				.06	.85
Baseline	23	34.05 (1.90)	30.24-37.86		26	33.74 (1.79)	30.16-37.31		24	35.22 (1.86)	31.50-38.94		
8 weeks	23	37.09 (1.99)	33.10-41.07		26	37.30 (1.87)	33.56-41.04		24	38.87 (1.95)	34.98-42.76		
3 months	23	38.87 (1.87)	35.14-42.60		26	39.53 (1.75)	36.03-43.03		24	39.78 (1.82)	36.14-43.43		
**WAIS-IV symbol coding**				.22				.001				.43	.35
Baseline	23	65.81 (3.54)	58.74-72.89		27	64.55 (3.26)	58.04-71.07		24	65.92 (3.47)	58.99-72.84		
8 weeks	23	73.80 (3.69)	66.43-81.18		27	70.22 (3.40)	63.43-77.01		24	73.39 (3.61)	66.18-80.60		
3 months	23	76.46 (3.69)	69.08-83.84		27	71.89 (3.40)	65.09-78.68		24	76.92 (3.61)	69.70-84.13		
**WAIS-IV cancellation**				.79				.23				.37	.60
Baseline	23	40.34 (2.12)	36.10-44.59		27	37.87 (1.96)	33.96-41.78		24	38.47 (2.08)	34.32-42.62		
8 weeks	23	42.52 (2.07)	38.38-46.66		27	39.53 (1.91)	35.72-43.34		24	42.43 (2.02)	38.38-46.48		
3 months	23	42.35 (2.09)	38.16-46.53		27	39.57 (1.93)	35.72-43.42		24	40.27 (2.05)	36.18-44.36		

[a]TMT: Trail Making Test.
[b]WAIS-IV: Wechsler Adult Intelligence Scale-Fourth Edition.
