# Supplementary material for: Digital Gaming for Improving the Functioning of People With Traumatic Brain Injury: Randomized Clinical Feasibility Study
Source: J Med Internet Res. 2018 Mar 19;20(3):e77. doi: 10.2196/jmir.7618 (PMC5881042; doi:10.2196/jmir.7618)
Supplement: Multimedia Appendix 2 [file jmir_v20i3e77_app2.pdf]

Secondary outcomes	CogniFit				PlayStation 3				Control				Time x Group interaction
	N	Mean (SE)	95% CI	*P* value	N	Mean (SE)	95% CI	*P* value	N	Mean (SE)	95% CI	*P* value	*P* value
**Attention and executive functions**													
**Simon task**				.20				.68				.36	.94
Baseline	24	41.77 (7.78)	57.29-26.24		26	49.70 (7.43)	64.54-34.87		23	49.15 (7.93)	64.98-33.31		
8 weeks	24	33.47 (6.03)	45.51-21.43		26	39.30 (5.76)	50.81-27.79		23	35.76 (6.15)	48.04-23.48		
3 months	24	36.56 (5.22)	46.98-26.14		26	41.94 (4.99)	51.90-31.98		23	35.82 (5.32)	46.45-25.20		
**Working memory**													
**WAIS-IV[a] digit span**				.26				.29				.60	.53
Baseline	23	26.45 (1.23)	23.98-28.92		26	26.13 (1.16)	23.81-28.44		24	25.71 (1.20)	23.30-28.12		
8 weeks	23	27.76 (1.32)	25.12-30.39		26	27.32 (1.23)	24.85-29.79		24	27.67 (1.29)	25.09-30.24		
3 months	23	28.42 (1.17)	26.07-30.77		26	27.09 (1.10)	24.88-29.29		24	26.70 (1.15)	24.40-29.00		
**PASAT[b] 3 s**				.85				.21				.22	.33
Baseline	20	45.91 (2.50)	40.90-50.92		24	43.45 (2.28)	38.87-48.02		22	46.44 (2.38)	41.67-51.22		
8 weeks	20	49.78 (2.30)	45.17-54.39		24	45.35 (2.10)	41.13-49.56		22	51.90 (2.19)	47.50-56.30		
3 months	20	51.61 (2.37)	46.87-56.36		24	47.57 (2.16)	43.24-51.90		22	52.85 (2.26)	48.33-57.38		
**PASAT 2 s**				.03				.35				.003	.67
Baseline	20	36.57 (2.14)	32.27-40.86		24	31.18 (1.96)	27.27-35.10		22	34.82 (2.04)	30.73-38.91		
8 weeks	20	39.52 (2.44)	34.63-44.41		24	33.81 (2.23)	29.35-38.28		22	38.81 (2.33)	34.15-43.47		
3 months	20	44.10 (2.53)	39.04-49.16		24	36.37 (2.31)	31.75-40.99		22	40.94 (2.41)	36.12-45.77		
**Depression**													
**Patient Health Questionnaire-9**				.05				.51				.84	.48
Baseline	23	5.04 (3.82)	3.39-6.70		25	4.12 (4.94)	2.08-6.16		22	6.36 (5.91)	3.74-8.98		
8 weeks	23	6.22 (5.30)	3.93-8.51		25	4.84 (4.97)	2.79-6.89		22	6.77 (6.47)	3.91-9.64		
3 months	23	6.65 (5.00)	4.49-8.82		25	4.28 (4.67)	2.35-6.21		22	6.46 (6.35)	3.64-9.27		

**Self-efficacy**													
**General Self-Efficacy Scale**				.61				.91				.06	.70
Baseline	23	30.30 (5.97)	27.72-32.89		25	30.04 (4.24)	28.29-31.79		22	29.64 (5.19)	27.34-31.94		
8 weeks	23	30.78 (5.58)	28.37-33.19		25	30.24 (4.30)	28.46-32.02		22	31.14 (5.29)	28.79-33.48		
3 months	23	31.13 (5.71)	28.66-33.60		25	29.92 (5.44)	27.68-32.17		22	30.73 (6.47)	27.86-33.60		
**Executive functions**													
**BRIEF-A[c]**				.08				.33				.13	.10
Baseline	20	108.6 (6.22)	96.17-121.1		20	114.7 (6.19)	102.3-127.1		21	121.9 (6.06)	109.8-134.1		
8 weeks	20	106.0 (6.00)	94.01-118.0		20	114.5 (5.97)	102.5-126.4		21	119.3 (5.84)	107.6-131.0		
3 months	20	110.4 (6.21)	97.99-122.8		20	107.2 (6.18)	94.85-119.6		21	120.8 (6.04)	108.7-132.9		

[a]WAIS-IV: Wechsler Adult Intelligence Scale-Fourth Edition.

[b]PASAT: Paced Auditory Serial Addition Test.

[c]BRIEF-A: Behavior Rating Inventory of Executive Functioning-Adult version.
