# Supplementary material for: Digital Gaming for Improving the Functioning of People With Traumatic Brain Injury: Randomized Clinical Feasibility Study
Source: J Med Internet Res. 2018 Mar 19;20(3):e77. doi: 10.2196/jmir.7618 (PMC5881042; doi:10.2196/jmir.7618)
Supplement: Multimedia Appendix 3 [file jmir_v20i3e77_app3.pdf]

Title of your manuscript *

Provide the (draft) title of your manuscript.

Digital Gaming for Improving the Functioning of People with Traumatic Brain Injury:

A Randomized Clinical Feasibility Study

TITLE AND ABSTRACT

1a) TITLE: Identification as a randomized trial in the title

1a) Does your paper address CONSORT item 1a? *

I.e does the title contain the phrase "Randomized Controlled Trial"? (if not, explain the reason under "other")

“Digital Gaming for Improving the Functioning of People with Traumatic Brain Injury:

A Randomized Clinical Feasibility Study”

Does your paper address subitem 1a-ii?

No non-web-based components, thus not addressed.

1a-iii) Primary condition or target group in the title
Does your paper address subitem 1a-iii? *
“..of People with Traumatic Brain Injury”

1b) ABSTRACT: Structured summary of trial design, methods, results, and conclusions
1b-i) Key features/functionalities/components of the intervention and comparator in the
METHODS section of the ABSTRACT
Does your paper address subitem 1b-i? *
“..one of three groups: a rehabilitation gaming group (..intervention group), an entertainment gaming group (..active control group) or a passive control group (n = 32). The gaming groups were instructed to engage in gaming for a minimum of 30 minutes per day for eight weeks.”

Does your paper address subitem 1b-ii?
No.

Does your paper address subitem 1b-iii?

“..were recruited from an outpatient neuroscience clinic, by telephone or by mail.”

“Cognitive measurements were conducted in face-to-face interviews by trained psychologists and questionnaires were self-administered.”

1b-iv) RESULTS section in abstract must contain use data

Does your paper address subitem 1b-iv?

“Participants did use the games (93% rehabilitation group, 100% entertainment group)… However, total time spent on gaming during the intervention period was low (15.22 h rehabilitation gaming group, 19.22 h entertainment gaming group).”

1b-v) CONCLUSIONS/DISCUSSION in abstract for negative trials

Does your paper address subitem 1b-v?

“We did not find differences between the groups in improvement in the outcome measures. The improvements in test performance by all three groups may reflect rehearsal effects.”

INTRODUCTION
2a) In INTRODUCTION: Scientific background and explanation of rationale

2a-i) Problem and the type of system/solution

Does your paper address subitem 2a-i? *

The problem, traumatic brain injury (TBI), is described related to how many people are affected globally, what are the problems caused by TBI (symptoms, burden for individuals and health systems). The solution, therapeutic rehabilitation interventions – specifically computer-based rehabilitation gaming and video-gaming – are described.

2a-ii) Scientific background, rationale: What is known about the (type of) system

Does your paper address subitem 2a-ii? *

What is known about computer-based rehabilitation gaming and video-gaming are described, use of the interventions in TBI and other patient groups (e.g. healthy adults, spinal cord injury, and stroke patients) are described. The motivation for the study is described:

“It is also still unknown if the benefits of video game training can be transferred to clinical settings and if games can improve cognitive functions important for management of daily activities. Furthermore, it would be prudent to explore whether gaming has any positive effects for patients with TBI. As TBI often results in long-term disability with adverse social, psychological and economic consequences, it is important to seek methods that optimize independence and social participation to reduce long-term care needs and enhance quality of life.”

2b) In INTRODUCTION: Specific objectives or hypotheses
Does your paper address CONSORT subitem 2b? *

“In this study, we aim to evaluate the effects and feasibility of digital games for improving cognitive functioning and well-being among people with TBI. We hypothesized, that among patients with TBI in the intervention group (rehabilitation

gaming), in comparison to the active control group (entertainment gaming) and passive control group, there would be a greater improvement in cognitive functioning (processing speed and visuomotor tasks, attention and executive functions, working memory) and well-being (depression, self-efficacy).”

METHODS

3a) Description of trial design (such as parallel, factorial) including allocation ratio

Does your paper address CONSORT subitem 3a? *

“The study includes a three arm, parallel and randomized clinical trial examining the effectiveness and feasibility of digital gaming for improving cognitive functioning and general well-being in people with TBI. The full study design and detailed description of the study methods can be found elsewhere [Välimäki et al. 2016 [46]],” where allocation ratio is explained based on power calculations (30 participants in each group, thus allocation ratio is 1:1:1).

3b) Important changes to methods after trial commencement (such as eligibility criteria), with reasons

Does your paper address CONSORT subitem 3b? *

No Important changes to methods after trial commencement.

3b-i) Bug fixes, Downtimes, Content Changes

Does your paper address subitem 3b-i?

No changes to trial methods due to bug fixes, downtimes or content changes.

4a) Eligibility criteria for participants

Does your paper address CONSORT subitem 4a? *

“The eligibility criteria stipulated that participants must be Finnish-speaking and reading adults, between 18 and 65 years old, who have been diagnosed with TBI (ICD-10, S06.X, T90.5), were discharged from the hospital at least 12 months before the recruitment, and who have had no active participation in cognitive rehabilitation during the last 3 months. In addition, eligible participants had to own a TV and a computer and have internet access at home. Active digital gamers were excluded, with the allowed gaming time being 5 hours or less per week [36].

Potential participants were excluded if they had sensory impairment (e.g. serious visual impairment), severe cognitive impairment (e.g. memory problems, slow processing speed, lack of attention, linguistic problems), a physical impairment that may restrict the use of computers or computer game control systems unaided (e.g. hemiplegia, dysfunction of the central vestibular system), apathy identified in previous neuropsychological evaluations, or a diagnosis of a severe mental disorder (e.g. schizophrenia or severe depressive disorders identified as the secondary diagnosis).”

4a-i) Computer / Internet literacy
Does your paper address subitem 4a-i?

No, because there was no criterion based on computer/internet literacy. However, the participants had to own a computer and have internet access at home: “In addition, eligible participants had to own a TV and a computer and have internet access at home”.

“Active digital gamers were excluded, with the allowed gaming time being 5 hours or less per week [36]”

4a-ii) Open vs. closed, web-based vs. face-to-face assessments:

Does your paper address subitem 4a-ii? *

“Patient eligibility was assessed primarily by reviewing Turku University Hospital electronic medical records, after which potentially eligible patients were further interviewed via telephone and assessed face-to-face by a trained psychologist.”

4a-iii) Information giving during recruitment
Does your paper address subitem 4a-iii?

“Those patients who were assessed to meet the inclusion criteria were contacted by telephone or by mail... Eligible participants with preliminary interest toward the study received written information about the study by mail … and a short description of the eight entertainment games (in case of allocation to the entertainment gaming group).”

“The study participants were informed orally (at least two telephone calls and one face-to-face meeting) and in written format of how and where their information was to be accessed, what the purpose of the study was and what specific steps to be taken were to be (if agreed to participate in the study).

4b) Settings and locations where the data were collected
Does your paper address CONSORT subitem 4b? *

“Cognitive tests were conducted in the research laboratory and self-administered questionnaires were sent to participants homes to be filled out before each visit to the research laboratory. Participants returned the questionnaires during the visit to the research laboratory.”

4b-i) Report if outcomes were (self-)assessed through online questionnaires

Does your paper address subitem 4b-i? *

“In the Patient Health Questionnaire (PHQ-9) [60,61], a self-administered questionnaire, respondents are asked to indicate..”

“The General Self-Efficacy Scale (GSC) [61] is a self-administered scale, which assesses a general sense of perceived self-efficacy..”

“The Behavior Rating Inventory of Executive Function-Adult Version (BRIEF-A) is a 75-item self-administrated questionnaire..”

4b-ii) Report how institutional affiliations are displayed

Does your paper address subitem 4b-ii?

N/A

5) The interventions for each group with sufficient details to allow replication, including how and when they were actually administered

5-i) Mention names, credential, affiliations of the developers, sponsors, and owners

Does your paper address subitem 5-i?

The researchers have no affiliations with the game (intervention) providers.

“Patients in the rehabilitation gaming group (intervention group) used an internet browser-based digital brain training program, CogniFit [47]”. The link to CogniFit-webpage is provided in references, where additional information can be found.

“Participants in the entertainment gaming group (active control group) used commercial digital games designed for Sony Playstation 3 (PS3) consoles”

5-ii) Describe the history/development process

Does your paper address subitem 5-ii?

The dose, frequency and length of the intervention, were based on previous research.

“The participants were guided to use the rehabilitation game for at least 30 minutes per day [34,50,51] over a period of eight weeks.”

5-iii) Revisions and updating

Does your paper address subitem 5-iii?

N/A

5-iv) Quality assurance methods

Does your paper address subitem 5-iv?

“Information about the frequency of training sessions was also recorded by the participants themselves in a gaming diary. The participants’ adherence to and motivation [49] for gaming were supported and monitored by weekly telephone calls. … during these telephone calls, participants had the possibility report any technical problems. To encourage, motivate, and hold participants to training, they were supported in planning a schedule for their training sessions (days, time, and frequency) for the entire eight-week gaming period.”

“A technical assistant was available to visit the participant’s home to help set up the console [52] or guidance by telephone.”

5-v) Ensure replicability by publishing the source code, and/or providing screenshots/screen-capture video, and/or providing flowcharts of the algorithms used

Does your paper address subitem 5-v?

The internet address of the intervention (Gognifit) is provided in the references (number 47).

5-vi) Digital preservation

Does your paper address subitem 5-vi?

The Cognifit-page is archived in webcitation.org.

5-vii) Access

Does your paper address subitem 5-vii? *

“A new email address, a password for the email account and a personal game account were generated for each participant, as the browser-based program required access through a website, and the user would log in with an email address and a specified password.”

“Participants in the entertainment gaming group (active control group) used commercial digital games designed for Sony Playstation 3 (PS3) consoles. The project purchased the participant-selected game (see below) from the official Playstation Store and downloaded and installed the game into the console given to the participant”

**5-viii) Mode of delivery, features/functionalities/components of the intervention and
comparator, and the theoretical framework**

Does your paper address subitem 5-viii? *

The dose, frequency and length of the intervention, were based on previous research.

“The participants were guided to use the rehabilitation game for at least 30 minutes per day [34,50,51] over a period of eight weeks.”

Intervention group:

“Patients in the rehabilitation gaming group (intervention group) used an internet browser-based digital brain training program, CogniFit [47]. We used a web-based cognitive training platform with 33 games designed with the purpose of improving the user's cognitive abilities as brain exercises. To ensure a user-centered approach, the participants were instructed to play at least one exercise from each of the three categories (memory, spatial perception, mental planning) during each training

session daily, and otherwise they were free to choose which exercises they wished to play."

"…written instructions for the rehabilitation game were given to the participant. In addition, instructions for the rehabilitation game were introduced to the participants during the introductory meeting, which took about 30 minutes per person. During the meeting, participants' abilities and previous experience in playing digital games were explored to ensure that the participants had the basic gaming skills required for active gaming.… and the user would log in with an email address and a specified password. The participant also tested the game unaided to find out possible barriers in their gaming…The participants' adherence to and motivation [49] for gaming were supported and monitored by weekly telephone calls.… The participant had also a possibility to monitor their own progress in the programme… To encourage, motivate, and hold participants to training, they were supported in planning a schedule for their training sessions (days, time, and frequency) for the entire eight-week gaming period".

Active control group:

"Participants in the entertainment gaming group (active control group) used commercial digital games designed for Sony Playstation 3 (PS3) consoles. The project purchased the participant-selected game (see below) from the official Playstation Store and downloaded and installed the game into the console given to the participant. Games to be selected by the participants (a total of eight games, see Figure 2) were considered to correspond to the rehabilitation games and to contain the same core gameplay elements [see 46]."

The games are presented in figure 2.

"Again, during the introductory meeting (about 30 minutes per person), written instructions regarding how to use the console were given to the participant, and the game the participant selected was tested with the researcher. As with the intervention group, ability to play digital games was explored to ensure that participants had the basic gaming skills required for active gaming. An overview of the use of the console was also offered and a tutorial demonstration was given (how to start the console, how to play the game, how to use the controller, how to change game options, such as game difficulty and speed, and so on). A technical assistant was available to visit the participant's home to help set up the console [52] or guidance by telephone. The participants were guided to play the console for at least 30 minutes per day over a period of eight weeks [34]. The participants were supported in planning their training session schedule (days and times), and information about game sessions (day, time, frequency, play progress) was recorded by the participant in a gaming diary. Therefore, participant was also able to monitor his/her own progress in the game.   Further, adherence to gaming was supported and monitored by weekly telephone calls."

5-ix) Describe use parameters

Does your paper address subitem 5-ix?

"..participants were instructed to play at least one exercise from each of the three categories (memory, spatial perception, mental planning) during each training

session daily, and otherwise they were free to choose which exercises they wished to play… The participants were guided to use the rehabilitation game for at least 30 minutes per day [34,50,51] over a period of eight weeks.”

“As in the intervention group, the participants chose an entertainment game that they found enjoyable.. However, the participants were not forced to play any one type of game, and they were able to change the game during the eight-week intervention period if they had concerns… The participants were guided to play the console for at least 30 minutes per day over a period of eight weeks”

5-x) Clarify the level of human involvement

Does your paper address subitem 5-x?

Rehabilitation gaming: “….instructions for the rehabilitation game were introduced to the participants during the introductory meeting with researcher (two different researchers, both registered nurses and Masters’ degree in Nursing Science), which took about 30 minutes per person …gaming were supported and monitored by weekly telephone calls. The telephone calls were made by two researchers and one research assistant. Researchers had qualifications of registered nurse and Masters’ degree in Nursing Science and the research assistant had a degree of Public Health Nurse and Bachelor’s degree in Nursing Science.”

Entertainment gaming: “…during the introductory meeting (about 30 minutes per person).. and the game the participant selected was tested with the researcher (two different researchers, both registered nurses and Masters’ degree in Nursing Science)… Further, adherence to gaming was supported and monitored by weekly telephone calls. The telephone calls were made by the same researchers and research assistant than in the rehabilitation gaming group”

5-xi) Report any prompts/reminders used

Does your paper address subitem 5-xi? *

Rehabilitation gaming: “…The participants’ adherence to and motivation [49] for gaming were supported and monitored by weekly telephone calls”

“Entertainment gaming: Further, adherence to gaming was supported and monitored by weekly telephone calls.”

5-xii) Describe any co-interventions (incl. training/support)

Does your paper address subitem 5-xii? *

“..written instructions for the rehabilitation game were given to the participant. In addition, instructions for the rehabilitation game were introduced to the participants during the introductory meeting with researcher …., which took about 30 minutes per person. During the meeting, participants’ abilities and previous experience in playing digital games were explored to ensure that the participants had the basic gaming skills required for active gaming... The participant also tested the game unaided to find out possible barriers in their gaming... The participants’ adherence to and motivation [49] for gaming were supported and monitored by weekly telephone calls”

“…during the introductory meeting (about 30 minutes per person), written instructions regarding how to use the console were given to the participant, and the game the participant selected was tested with the researcher.. An overview of the use of the console was also offered and a tutorial demonstration was given (how to start the console, how to play the game, how to use the controller, how to change game options, such as game difficulty and speed, and so on). A technical assistant was available to visit the participant’s home to help set up the console [52] or guidance by telephone… The participants were supported in planning their training session schedule (days and times).. To encourage, motivate, and hold participants to training, they were supported in planning a schedule for their training sessions (days, time, and frequency) for the entire eight-week gaming period”

6a) Completely defined pre-specified primary and secondary outcome measures, including how and when they were assessed

Does your paper address CONSORT subitem 6a? *

Primary outcome: Processing Speed and Visuomotor Tasks (Trail Making Test (TMT):

TMT A, TMY B)

Secondary outcomes: Attention and Executive Functions (The Simon task), Working

Memory (WAIS-IV, PASAT), Depression (PHQ-9), Self-efficacy (GSE), Executive

Functions (BRIEF-A)

“Patient data were collected at three different times: at baseline, after the intervention (eight weeks, between September 2015 and December 2015), and three months after the intervention ended (between December 2015 and April 2016).”

“..cognitive measurements were conducted by a trained psychologist at the test laboratory”

“Cognitive tests were conducted in the research laboratory and self-administered questionnaires were sent to participants homes to be filled out before each visit to the research laboratory. Participants returned the questionnaires during the visit to the research laboratory.”

“….received written information about the study by mail in addition to informed consent forms, baseline questionnaires to be filled out”

6a-i) Online questionnaires: describe if they were validated for online use and apply CHERRIES items to describe how the questionnaires were designed/deployed

Does your paper address subitem 6a-i?

No online questionnaires used.

6a-ii) Describe whether and how "use" (including intensity of use/dosage) was defined/measured/monitored

Does your paper address subitem 6a-ii?

Rehabilitation gaming: "Information about the frequency of training sessions was also recorded by the participants themselves in a gaming diary"

Entertainment gaming: "...and information about game sessions (day, time, frequency, play progress) was recorded by the participant in a gaming diary".

"The gaming information concerning rehabilitation gaming group were collected from game logs retrieved from the gaming system. Regarding entertainment games, the information was collected from console gaming logs where possible and from the gaming diaries where the logs were not available."

6a-iii) Describe whether, how, and when qualitative feedback from participants was obtained
Does your paper address subitem 6a-iii?

"The participants had a possibility to specify their answers by answering to open ended questions (not analyzed in the study due to limited size of the data)."

6b) Any changes to trial outcomes after the trial commenced, with reasons

Does your paper address CONSORT subitem 6b? *

No changes to trial outcomes after the trial commencement.

7a) How sample size was determined

NPT: When applicable, details of whether and how the clustering by care provides or centers was addressed

7a-i) Describe whether and how expected attrition was taken into account when calculating the sample size
Does your paper address subitem 7a-i?

"Based on our preliminary power calculations [see 46], the sample size was expected to be 30 in each group, which is not very strong but reasonable enough for a feasibility study aiming to detect changes within a group between baseline and follow-up outcome measurements with an expected attrition rate close to 0%."

From the study protocol [46, Välimäki et al. 2016]: The calculations for the sample size needed in each group are preliminary estimations to guide our data collection, which are based on previous studies: (1) TMT (version A) and (2) depression (PHQ-9). First, if a score on the TMT version will be about 71, the mean change in the scores during the follow-up will be 30, and standard deviation of the TMT scores will be 53 [41]. This difference between groups could be expected to be significant (with a

power of 85%, p=.05) if the sample size in each group is 30 subjects. Second, if the average level of the PHQ-9 score is about 10, the mean change in the scores during the follow-up is then 3 (SD 5) [48]. The difference could be expected to be significant (with a power of 85%, p=.05) if the sample size in each group is 27 subjects. Thus, based on these preliminary power calculations, the sample size to be used in this study (30 in each group) is not very strong but will be reasonable for a feasibility study aiming to detect preliminary changes within the group, between baseline and follow-up outcome measurements. However, this means that the attrition rate of the study should be near 0%.

7b) When applicable, explanation of any interim analyses and stopping guidelines

Does your paper address CONSORT subitem 7b?

N/A

8a) Method used to generate the random allocation sequence

NPT: When applicable, how care providers were allocated to each trial group

Does your paper address CONSORT subitem 8a?

The method used to generate the allocation sequence was computer generated:

”.. randomly assigned … the participants using randomization software (SAS for Windows, Version 9.3).”

8b) Type of randomisation; details of any restriction (such as blocking and block size)

Does your paper address CONSORT subitem 8b? *

“a block randomization in three blocks”

9) Mechanism used to implement the random allocation sequence (such as sequentially numbered containers), describing any steps taken to conceal the sequence until interventions were assigned

Does your paper address CONSORT subitem 9? *

“An independent trial statistician outside the study group randomly assigned … the participants.. The randomization list was delivered to the trial manager outside the study group.”

“…If the contacted individual was interested in participating, the trial manager then received a message (by email, text message or telephone) sent by the recruiting researcher.”

“The trial manager informed the researchers about participants’ group after the baseline assessments.”

10) Who generated the random allocation sequence, who enrolled participants, and who assigned participants to interventions

Does your paper address CONSORT subitem 10?

“An independent trial statistician outside the study group randomly assigned … the participants..”. The randomization and patient allocation were fully centralized (at the University of Turku).

“Those patients who were assessed to meet the inclusion criteria were contacted by telephone or by mail between 22 June and 24 November 2015 by researchers”

If the contacted individual was interested in participating, the trial manager then received a message (by email, text message or telephone) sent by the recruiting researcher

11a) If done, who was blinded after assignment to interventions (for example, participants, care providers, those assessing outcomes) and how

NPT: Whether or not administering co-interventions were blinded to group assignment

11a-i) Specify who was blinded, and who wasn’t

Does your paper address subitem 11a-i? *

“The researchers overseeing patient recruitment and randomization were therefore aware of the assignments. Due to the intervention type, allocation was not masked to participants in the intervention and control groups or to researchers who recruited patients. The psychologists, as cognitive outcome assessors, were kept blinded. However, in some occasions study participants told them about their possible game playing. The data analyst (the trial statistician) was kept blinded to the allocation..”

11a-ii) Discuss e.g., whether participants knew which intervention was the “intervention of interest” and which one was the “comparator”

Does your paper address subitem 11a-ii?

The participants were aware of the intervention of interest.

“Due to the intervention type, allocation was not masked to participants”

11b) If relevant, description of the similarity of interventions

Does your paper address CONSORT subitem 11b? *

Entertainment gaming group (active control group):
“Games to be selected by the participants (a total of eight games, see Figure 2) were considered to correspond to the rehabilitation games and to contain the same core gameplay elements [see 46]”

12a) Statistical methods used to compare groups for primary and secondary outcomes

Does your paper address CONSORT subitem 12a? *

“The data were analyzed with a Repeated Measures ANOVA, in which Group (rehabilitation gaming, entertainment gaming, and passive control) was a between-subjects factor and Time (before intervention, after intervention, and follow-up) was a within-subjects factor. Effectiveness of the intervention was indicated by a significant Group*Time interaction, which indicated differences between the three groups in the improvement of the primary and secondary outcomes over time.”

12a-i) Imputation techniques to deal with attrition / missing values

Does your paper address subitem 12a-i? *

“For sensitivity analysis, we calculated the effect sizes for each participant (ITT). We also performed analyses on both completer-only data and compared the study results between these two groups. However, no differences between the results were found in ITT analysis or for those completing the follow-ups. “

12b) Methods for additional analyses, such as subgroup analyses and adjusted analyses

Does your paper address CONSORT subitem 12b? *

“Repeated measures ANOVAs were also performed for each group separately.”

X26) REB/IRB Approval and Ethical Considerations [recommended as subheading under "Methods"] (not a CONSORT item)

X26-i) Comment on ethics committee approval

Does your paper address subitem X26-i?

“The study was evaluated by the Ethics Committee of the Turku University Hospital (ETMK 41/1801/2015)”

x26-ii) Outline informed consent procedures

Does your paper address subitem X26-ii?

“The study participants were informed orally (at least two telephone calls and one face-to-face meeting) and in written format of how and where their information was to be accessed, what the purpose of the study was and what specific steps to be

taken were to be (if agreed to participate in the study). Written informed consent was obtained in accordance with the Declaration of Helsinki [63].”

“Eligible participants were then invited to the research laboratory, at which time an informed consent form was signed.”

X26-iii) Safety and security procedures

Does your paper address subitem X26-iii?

“To identify any ethical or practical concerns in the study protocol, entertainment and rehabilitation games were pre-tested with five healthy adults and with five people with TBI. Based on pre-tests, more specific inclusion and exclusion criteria for the study were identified. In addition, some games initially identified to be used in the study were excluded if they were suspected to cause dizziness or headaches due to dark colors or 3D tunnel effects [64].”

RESULTS

13a) For each group, the numbers of participants who were randomly assigned, received intended treatment, and were analysed for the primary outcome

Does your paper address CONSORT subitem 13a? *

The numbers of participants who were randomly assigned, received intended treatment: Figure 1 Participants who were analyzed for the primary outcome: Table 2.

13b) For each group, losses and exclusions after randomisation, together with reasons

Does your paper address CONSORT subitem 13b? (NOTE: Preferably, this is shown in a CONSORT flow diagram) *

Yes, for each group, losses and exclusions after randomisation, together with reasons are presented in Figure 1, CONSORT flow diagram and as text in Results, under heading “Sample characteristics”.

13b-i) Attrition diagram

Does your paper address subitem 13b-i?

No diagram or table.

“During the eight-week intervention period, the average gaming time in the entertainment gaming group was 19.22 hours (range 0-71.48 hours) and in the rehabilitation gaming group 15.02 hours (range 0.12-71.38 hours).”

14a) Dates defining the periods of recruitment and follow-up

Does your paper address CONSORT subitem 14a? *

“The hospital electronic medical records were accessed (June 2015), and patients with a TBI diagnosis were screened to determine which patients fulfilled the eligibility criteria for study participation. Those patients who were assessed to meet the inclusion criteria were contacted by telephone or by mail between 22 June and 24 November 2015”

“Patient data were collected at three different times: at baseline, after the intervention (eight weeks, between September 2015 and December 2015), and three months after the intervention ended (between December 2015 and April 2016).”

14a-i) Indicate if critical “secular events” fell into the study period

Does your paper address subitem 14a-i?

N/A

14b) Why the trial ended or was stopped (early)

Does your paper address CONSORT subitem 14b? *

The trial was not stopped early.

15) A table showing baseline demographic and clinical characteristics for each group

Does your paper address CONSORT subitem 15? *

Detailed characteristics of the participants are described in Table 1.

15-i) Report demographics associated with digital divide issues

Does your paper address subitem 15-i? *

Detailed characteristics of the participants are described in Table 1.

16) For each group, number of participants (denominator) included in each analysis and whether the analysis was by original assigned groups

16-i) Report multiple “denominators” and provide definitions

Does your paper address subitem 16-i? *

Yes. Numbers of each participant included in analysis can be found in tables 1-3. Participants who received intervention in intervention group and active control group can be found in figure 1.

16-ii) Primary analysis should be intent-to-treat

Does your paper address subitem 16-ii?

“For sensitivity analysis, we calculated the effect sizes for each participant (ITT). We also performed analyses on both completer-only data and compared the study results between these two groups. However, no differences between the results were found in ITT analysis or for those completing the follow-ups.”

17a) For each primary and secondary outcome, results for each group, and the estimated effect size and its precision (such as 95% confidence interval)

Does your paper address CONSORT subitem 17a? *

Yes. Effect sizes (F-values) regarding are presented in the results section as text related to each of the outcomes. Their precision (95% CI) is presented in tables 2 (primary outcome) and 3 (secondary outcome).

17a-i) Presentation of process outcomes such as metrics of use and intensity of use

Does your paper address subitem 17a-i?

During the eight-week intervention period, the average gaming time in the entertainment gaming group was 19.22 hours (range 0-71.48 hours) and in the rehabilitation gaming group 15.02 hours (range 0.12-71.38 hours).

17b) For binary outcomes, presentation of both absolute and relative effect sizes is recommended

Does your paper address CONSORT subitem 17b? *

No binary outcomes in the paper.

18) Results of any other analyses performed, including subgroup analyses and adjusted analyses, distinguishing pre-specified from exploratory

Does your paper address CONSORT subitem 18? *

Yes. The results of Repeated measures ANOVAs for each group separately are presented as text in results section and in tables 2 (primary outcome) and 3 (secondary outcomes).

1 Does your paper address subitem 18-i?

18-i) Subgroup analysis of comparing only users

Not conducted due to limited sample size.

19) All important harms or unintended effects in each group

(for specific guidance see CONSORT for harms)

Does your paper address CONSORT subitem 19? *

Regarding depression: "On the contrary, in the rehabilitation gaming group, the mean scores increased from baseline to eight weeks and from baseline to three months (Mean 5.0 [SD 3.8] to Mean 6.7 [SD 5.00], P = .048), showing increase in the participants' depressive symptoms. Even so, on the categorical level (mild vs. moderate depression), the change observed between time-points was not clinically significant (scoring 6-9 points indicates minimal symptoms, UMHS Depression Guideline, August 2011)."

19-i) Include privacy breaches, technical problems

Does your paper address subitem 19-i?

There were three different changes/problems to rehabilitation gaming intervention due to system updates, that were not dependent of the research group.

The changes/problems occurred between 20.11.2015-1.12.2015 and they affected the playing of 13 participants, and were as follows:

1) pre-specified gaming categories (memory, spatial perception, mental planning) in Cognifit were not included in the participants user account (this was corrected after two days)
2) participant had to complete games in memory-category, before the participant could move on to games in other categories
3) the participant had to wait 1 hour after completing the games in one pre-specified category, before the participant was able to start playing games in other categories (20.11.2015-1.12.2015)

" Also, some functions in participants' rehabilitation game user accounts changed (e.g. pre-specified gaming categories in Cognifit were not included in the participants user account) for a short amount of time due to system updates by Cognifit. However, these changes affected only13 participants during 11 days, and therefore it is unlikely that they have affected the results."

19-ii) Include qualitative feedback from participants or observations from staff/researchers

Does your paper address subitem 19-ii?

"The participants had a possibility to specify their answers by answering to open ended questions (not analyzed in the study due to limited size of the data)."

DISCUSSION

22) Interpretation consistent with results, balancing benefits and harms, and considering other relevant evidence

22-i) Restate study questions and summarize the answers suggested by the data, starting with primary outcomes and process outcomes (use)

Does your paper address subitem 22-i? *

“In this study, we aimed to evaluate the effects and feasibility of digital games for improving cognitive functioning and well-being among people with TBI. We found no differences between the control group and the two intervention groups for the primary outcomes (processing speed and visuomotor tasks) or any of the secondary outcomes. Test scores improved in all groups over time regarding several different variables.”

22-ii) Highlight unanswered new questions, suggest future research

Does your paper address subitem 22-ii?

“In the future, a single game might be a better option in RCT design to ensure accuracy of the content of the different interventions.”

“Therefore, the participants’ own perceptions toward gaming and its use as part of rehabilitative interventions should be explored in more detail. In the future, patients could potentially be prescribed personalized gaming interventions based on specific cognitive deficits and their personal game preferences, which would improve the effectiveness of the intervention... In future studies, a more robust research design with a larger sample size is needed.”

20) Trial limitations, addressing sources of potential bias, imprecision, and, if relevant, multiplicity of analyses

20-i) Typical limitations in ehealth trials

Does your paper address subitem 20-i? *

“Low fidelity in the intervention may be a result of participants’ poor motivation to participate in the gaming intervention independently at home.”

“Second, we are unaware of how many participants in the control group were engaged in gaming activities, a factor that could positively affect their cognitive status”

Keeping these factors that may have affected the results in mind, this study has other limitations that should be taken into account. In the entertainment gaming group, the participants were given the chance to select their favorite game or change the game during the intervention. While all games included in this study were considered to contain similar game dynamics assumed to improve certain cognitive functions, there was some variability between the games, and it is possible that the game dynamics of the participant’s favorite game did not target the specific cognitive deficits of that participant. The choice of eight games also makes it difficult to conclude which types of game dynamics actually improve the cognitive functions of interest.

The games used in the present study may have also included too many action games, while participants might prefer other types of games..”

Some incidental factors could also have shown to affect the outcomes of the results, such as patient perceptions or attitudes toward gaming.

Finally, the sample size of the study was small, making it difficult to detect small effects (i.e., differences between groups), especially as the sample included a relatively heterogeneous group of patients with a wide variety of cognitive deficits.”

21) Generalisability (external validity, applicability) of the trial findings

21-i) Generalizability to other populations

Does your paper address subitem 21-i?

Finally, the sample size of the study was small, making it difficult to detect small effects (i.e., differences between groups), especially as the sample included a relatively heterogeneous group of patients with a wide variety of cognitive deficits. These factors limit the generalization of the results to a wider population.

21-ii) Discuss if there were elements in the RCT that would be different in a routine application setting

As far as we are aware, technological solutions are not used routinely in outpatient care for persons with TBI. Does your paper address subitem 21-ii?

OTHER INFORMATION
23) Registration number and name of trial registry

Does your paper address CONSORT subitem 23?

Trial Registration: Clinicaltrials.gov NCT02425527

24) Where the full trial protocol can be accessed, if available

Does your paper address CONSORT subitem 24? *

46. Välimäki M, Korkeila J, Kauppi K, Kaakinen JK, Holm S, Vahlo J, Tenovuo O, Hämäläinen H, Sarajuuri J, Rantanen P, Orenius T, Koponen A. Digital Gaming for Improving the Functioning of People With Traumatic Brain Injury: Protocol of a Feasibility Study. JMIR Res Protoc 2016 Feb 9;5(1):e6. PMID: 26860741

25) Sources of funding and other support (such as supply of drugs), role of funders

Does your paper address CONSORT subitem 25? *

“We would also like to show our appreciation to the partners who support the project, including The Finnish Funding Agency for Technology and Innovation (TEKES), The Finnish Association of People with Physical Disabilities, Oy Nordisk Film Ab/Playstation, TribeFlame Oy, BCB Medical, Validia Rehabilitation Helsinki, ORTON

Ltd, ORTON Foundation, Serious Games Finland Oy, the Hospital District of Southwest Finland, and Turku University Hospital.”

X27) Conflicts of Interest (not a CONSORT item)

X27-i) State the relation of the study team towards the system being evaluated

Does your paper address subitem X27-i?

“None declared.”
